# Draft genome sequence of *Cupriavidus* sp. strain TKC, isolated from a γ-hexachlorocyclohexane-degrading community

**DOI:** 10.1128/MRA.00567-23

**Published:** 2023-11-08

**Authors:** Hiromi Kato, Yoshiyuki Ohtsubo, Shoko Hirano, Sachiko Masuda, Arisa Shibata, Ken Shirasu, Yuji Nagata

**Affiliations:** 1 Institute for Agro-Environmental Science, National Agriculture and Food Research Organization, Kan-nondai, Tsukuba, Ibaraki, Japan; 2 Graduate School of Life Sciences, Tohoku University, Katahira, Sendai, Japan; 3 Plant Immunity Research Group, RIKEN Center for Sustainable Resource Science, Tsurumi-ku, Yokohama, Kanagawa, Japan; University of Southern California, Los Angeles, California, USA

**Keywords:** hexachlorocyclohexane, *Cupriavidus*

## Abstract

*Cupriavidus* sp. strain TKC was isolated from a microbial community enriched with γ-hexachlorocyclohexane (γ-HCH). This strain did not show γ-HCH-degrading activity but was one of the major members of the community. Here, we present the draft genome sequence of the strain TKC with a size of 7 Mb.

## ANNOUNCEMENT

Gamma isomer of hexachlorocyclohexane (γ-HCH) is a chlorinated organic insecticide that has caused environmental pollution because of its recalcitrant and toxic characteristics. A sludge sample with an aqueous layer was collected from an HCH-isomer-contaminated site on Kyushu Island, Japan (1, 2) and cultured at 30°C in 1/10 W minimum liquid medium (3) with γ-HCH as the sole carbon source to enrich HCH-degrading bacteria. After repeated single-colony-isolation on the same minimum agar plate containing γ-HCH, which made the plate cloudy, and 1/3 LB (3) agar plate, we finally obtained *Sphingobium* sp. strain TKS that showed HCH-degrading activity based on clear zone on the HCH agar plate (3) and several strains that did not form a clear zone (referred to as “non-degrading bacteria”). The genomes of the HCH-degrading strain TKS (2) and the non-degrading *Pseudomonas* sp. strain TKP (1) were previously reported. Here, we reported the draft genome of another non-degrading *Cupriavidus* sp. TKC. TKC cells grown on the 1/3 LB agar plate were used for DNA extraction by Marmur method (4) with modifications, in which we used TESS buffer containing 30 mM Tris-HCl, 5 mM EDTA, 50 mM NaCl, 25% sucrose, 5 mg/mL lysozyme, and protinase-K for cell lysis. For PacBio sequencing, the extracted DNA sample was shared with Megaruptor (Diagenode) on a 20-kb setting, size-selected (<12 kb cutoff) using BluePippin (Sage), and used for library preparation with SMRTbell Template Prep Kit 1.0-SPv3 (Pacific Biosciences). The exact same DNA extraction sample for PacBio was enzymatically shared and used for Illumina library preparation (insert size: 350 bp) with NEBNext Ultra II DNA Library Prep Kit (New England Biolabs). PacBio Sequel SMRTCell 1M v2 and Illumina Hiseq X sequencing generated 242,578 reads (average 8.2 kb, N50 11 kb) and 6,645,496 paired-end reads (150 b), respectively, and total length was 4.0 Gb (566-fold coverage). Raw reads were error corrected and assembled with Unicycler version 0.4.8 (5) in conservative mode and generated 18 contigs. Default parameters were used for all software unless otherwise specified. Sequence variations among repeat regions in the genome were corrected by three software (6); GenoFinisher ver. 3.04, AceFileViewer ver. 1.65, and ShortReadManager ver. 1.07, in which the Illumina reads were processed to trim the portion of reads with low k-mer abundance (<30 times), and reads longer than 45 b were used. A total length of the genome was 7,014,108 bp, with a G + C content of 63.6%. The longest contig length and N50 were both 3,735,270 bp. Annotation with DFAST version 1.4.0 (7, 8) detected 6,394 coding sequences, 68 tRNAs, and four rRNA operons. NCBI-blastn of its 16S rRNA showed 99% similarity to *Cupriavidus metallidurans* CH34 (9). Two-way average nucleotide identity (10, 11) against CH34 (GCF_000196015.1) was 96.6%. Homology analysis based on Kyoto Encyclopedia of Genes and Genome (12) did not detect γ-HCH-degrading genes (2). These data may provide insights into the co-operative mechanisms in HCH-degrading community.

## Data Availability

The sequence of 16S rRNA (Accession number: LC771448), the draft genome (BPWD01000001-BPWD01000018), and the raw data of Illumina (DRR489142) and PacBio (DRR489143) were deposited at DDBJ under Bioproject (PRJDB12339) with Biosample (SAMD00407105).
